# Construction of molecular identity card for cultivated *Dendrobium officinale* in Guizhou Province

**DOI:** 10.7717/peerj.21558

**Published:** 2026-07-28

**Authors:** Deqiang Ren, Changya Chen, Tinglu Wang, Xia Meng, Yuan Huang, Ping Wu

**Affiliations:** 1 Guizhou University of Traditional Chinese Medicine, Guiyang, Guizhou Provence, China; 2 Sichuan Academy of Chinese Medicine Sciences, Chengdu, Sichuan Provence, China

**Keywords:** Cultivated variety, *Dendrobium officinale*, SSR molecular markers, Molecular identity card, Construction

## Abstract

To establish a molecular identity card for *Dendrobium officinale*, providing a scientific basis for the breeding, utilization, standardized production, and quality traceability of cultivated varieties in Guizhou Province. Based on the published genome sequence of *Dendrobium officinale*, Simple Sequence Repeat (SSR) loci were identified using MISA (MicroSatellite identification tool) software, and SSR molecular markers were designed *via* Primer 3. Randomly selected primers were used for genetic analysis of cultivated *Dendrobium officinale* from Guizhou. Amplified fragments were digitally encoded and combined with supplementary information such as origin to construct a Quick Response (QR) molecular identity card. A total of 1,361,061 SSR markers were developed, with an average of 136 per scaffold. Mononucleotide repeats were the dominant motif (63.55%), among which (A) and (T) types accounted for 95.85%. Dinucleotide repeats constituted 11.62% of all SSR loci. Among 40 randomly selected primers, 27 were polymorphic, generating 127 polymorphic bands with a polymorphism rate of 96.27%. Genetic diversity analysis revealed rich genetic diversity in the tested materials. Cluster analysis divided the samples into four groups, based on which a molecular identity card for Guizhou cultivated *Dendrobium officinale* was successfully constructed. The SSR markers developed in this study exhibit high polymorphism and are suitable for genetic analysis of *Dendrobium officinale*. The constructed molecular identity card is of significant importance for ensuring the healthy development of the *Dendrobium officinale* industry in Guizhou.

## Introduction

*Dendrobium officinale* Kimura et Migo is a precious medicinal herb in China, recognized as both a medicine and food, and ranks first among the “Nine Rare Immortal Herbs” (Huang, Wang & Nie, 2023). It is the species with the highest economic value in the genus *Dendrobium*, with broad market prospects in healthcare applications (Hu et al., 2024). Almost all *Dendrobium officinale* on the market today is artificially cultivated. Guizhou Province is a major cultivation region with the largest area of simulated wild cultivation in China. However, alongside rapid industrial development, problems such as cultivar confusion and complex origins of introduced germplasm have become prominent. Most seedlings in Guizhou are introduced from Zhejiang, Yunnan, and Fujian, alongside locally domesticated wild materials. As a special commodity, medicinal plant seedlings cannot be reliably identified by ordinary growers based on morphology alone in terms of genetic origin or geographical source. Moreover, the content of active constituents varies considerably among different cultivated varieties (Ding et al., 2005), posing challenges to medication safety.

Establishing a molecular identity card for cultivated *Dendrobium officinale* in Guizhou, a digital DNA profile based on molecular markers that can distinguish individuals (Wang et al., 2025), would enable rapid, accurate germplasm identification, variety labeling, and traceability, thereby supporting breeding programs and standardized cultivation (Mao et al., 2024). Among the various molecular marker techniques used in medicinal plant genetics (Wang et al., 2024), SSR (Simple Sequence Repeat) markers, also known as microsatellites, are tandemly repeated motifs of 1–6 base pairs that are highly polymorphic, codominant, and widely distributed across eukaryotic genomes. Their stability across developmental stages and environmental conditions makes them particularly advantageous over morphological or biochemical markers. Consequently, SSR markers are increasingly applied in genetic diversity analysis, genetic linkage map construction, and QTL mapping in *Dendrobium officinale* (Zhou et al., 2023).

Despite these advances, a critical research gap remains: existing SSR marker studies in *Dendrobium officinale* have predominantly relied on markers derived from transcriptomes or limited genomic regions, resulting in uneven distribution of polymorphic primers across the genome. For instance, Xie et al. (2010) developed four SSR primer pairs for germplasm purity identification, while Xu et al. (2015) identified 249 SSR loci across 36 accessions and reported high genetic diversity. However, in both studies, polymorphic primers were unevenly distributed, with fewer polymorphic markers in certain chromosomal regions—likely due to their genomic locations (*e.g.*, telomeres, centromeres, conserved regions). This uneven distribution limits the accuracy of subsequent genetic analyses and genetic map construction. A genome-wide approach to SSR primer design is therefore essential for precise genetic characterization of this species.

To address this gap, the present study utilizes the published *Dendrobium officinale* genome to screen genome-wide polymorphic loci using MISA (Microsatellite Identification Tool) and to design high-throughput SSR primers *via* Primer 3. These markers will be used to analyze the genetic diversity of cultivated *Dendrobium officinale* in Guizhou Province and to construct molecular identity cards based on molecular fingerprinting. This research aims to provide a scientific basis for the breeding, utilization, and standardized cultivation of *Dendrobium officinale* in Guizhou.

## Materials & Methods

### Experimental materials

The samples used in this study were collected from cultivation sites across various counties and districts in Guizhou Province, with primary origins including Yunnan, Zhejiang, and Guizhou. All materials were authenticated as *Dendrobium officinale* Kimura et Migo by Shenghua Wei (Guizhou University of Traditional Chinese Medicine). The materials are deposited and maintained in the Germplasm Resource Nursery of Guizhou University of Traditional Chinese Medicine (Table 1).

**Table 1:** Germplasm resources of *Dendrobium officinale* used in this study.

	Accession ID	Provenance	Geographic origin	Cultivation method
1	ZJ-1	Zhejiang variety, with unknown breed	Guizhou Anlong Xicheng Xiushu Agriculture and Forestry Co., Ltd.	Imitation wild cultivation
2	ZJ-2	Zhejiang variety, species unknown	Guizhou Anlong Xicheng Xiushu Agriculture and Forestry Co., Ltd.	Facility cultivation
3	AL-1	Locally domesticated wild species in Anlong County, Guizhou Province	*Dendrobium* Garden Farmers’ Professional Cooperative, Pojiao Township, Anlong County, Guizhou Province	Facility cultivation
4	ZJ-3	Zhejiang variety, species unknown	Xicheng Xiushu Agriculture and Forestry Co., Ltd., Anlong County, Guizhou Province	Imitation wild cultivation
5	FJ-1	Fujian species, variety unknown	Guizhou Lvjian Shennong Organic Agriculture Co., Ltd., Dushan County, Guizhou Province	Facility cultivation
6	GZ-1	Guizhou Province Guihu No. 1	Guizhou Xingqian Technology Co., Ltd., Guiyang City, Guizhou Province	Facility cultivation
7	ZJ-4	No. 1, Senshan, Zhejiang Province	Guizhou Xingqian Technology Co., Ltd., Guiyang City, Guizhou Province	Facility cultivation
8	ZJ-5	Zhejiang variety, species unknown	Huazhai Domestication Base, Jinping County, Guizhou Province	Imitation wild cultivation
9	ZJ-6	Zhejiang variety, specific variety unknown	Guizhou Jinping Qianle *Dendrobium officinale* Planting Farmers’ Professional Cooperative, Jinping County, Guizhou Province	Imitation wild cultivation
10	YN-1	*Dendrobium* from Menghai, Yunnan Province	Libo Gaoli Aquaculture Production and Marketing Professional Cooperative, Libo County, Guizhou Province	Imitation wild cultivation
11	YN-2	Yunnan species, variety unknown	Hanlong Yuhu Biotechnology Co., Ltd., Luodian County, Guizhou Province	Imitation wild cultivation
12	LD-1	Locally domesticated wild species in Luodian County, Guizhou Province	Professional Cooperative for Planting in the Stone Mountain Area under Forests, Luodian County, Guizhou Province	Imitation wild cultivation
13	YN-3	Soft-footed *Dendrobium officinale* from Guangnan County, Yunnan Province	Guizhou Panxian Jinzhiyuan Traditional Chinese Medicine Planting Farmers’ Cooperative	Facility cultivation
14	ZJ-7	Zhejiang variety, with unknown species	Guizhou Wanhuyuan Ecological Agriculture Development Co., Ltd., Pingtang County, Guizhou Province	Facility cultivation
15	YN-4	Lijiang County, Yunnan Province, variety unknown	Guizhou Pingtang Huishengyuan Agriculture and Forestry Development Co., Ltd.	Facility cultivation
16	YN-5	Yunnan species, variety unknown	Guizhou Pingtang Huishengyuan Agriculture and Forestry Development Co., Ltd.	Facility cultivation
17	YN-6	Yunnan Guangnan County Soft-leg *Dendrobium officinale*	Guizhou Pingtang Huishengyuan Agriculture and Forestry Development Co., Ltd.	Facility cultivation
18	YN-7	Yunnan Guangnan Soft-leg *Dendrobium officinale*	Guizhou Qianxi Bohui Traditional Chinese Medicine Planting Professional Cooperative	Facility cultivation
19	ZJ-8	Yandang Red, Zhejiang Province	Guizhou Shuicheng Lingtouyan Seed Industry Technology Development Company	Facility cultivation
20	YN-8	No. 1, Xilin, Yunnan Province	Guizhou Bailing Enterprise Group Pharmaceutical Co., Ltd. (Bailing Ecological Park), Zhenning County, Guizhou Province	Facility cultivation

### Primer design and genotyping

Genomic DNA of *Dendrobium officinale* was extracted and subsequently detected *via* gel electrophoresis following the protocol described by Pan et al. (2023). To develop genome-wide SSR markers, the published *Dendrobium officinale* genome (NCBI assembly accession: ASM160598v2, GCA_001605985.2. The website is https://www.ncbi.nlm.nih.gov/datasets/genome/GCF_001605985.2/) was utilized. The MISA (MicroSatellite identification tool; http://pgrc.ipk-gatersleben.de/misa/) software was employed to screen for polymorphic microsatellite loci across the genome. High-throughput primer design was performed using Primer 3 (http://primer3.ut.ee/). The synthesized primers were provided by Sangon Biotech (Shanghai) Co., Ltd. The design parameters were as follows: primer length of 18–25 bp, expected PCR product size of 100–250 bp, and GC content of 40–60%. The Misa software configuration file, Perl script, and Genome-wide SSR molecular marker developed in this research (Table S1) have been uploaded to the science database (DOI: 10.57760/sciencedb.34283).

The polymerase chain reaction (PCR) was conducted in a total volume of 10 µL, containing five µL of Taq Master Mix, one µL (approximately 50 ng) of template DNA, one µL (10 µM) of SSR primer, and three µL of ddH_2_O. The amplification program consisted of an initial denaturation at 95 °C for 5 min; followed by 35 cycles of denaturation at 95 °C for 30 s, annealing at 50–60 °C (temperature adjusted based on the primer’s Tm value, typically 55–60 °C) for 45 s, and extension at 72 °C for 60 s; with a final extension at 72 °C for 10 min. If initial SSR-PCR amplification results were suboptimal, adjustments to the reaction components and/or annealing temperature were made to establish the optimal protocol.

Amplification products were separated by agarose gel electrophoresis on 1.2% agarose gels using 1 × TAE as the running buffer. Electrophoresis was performed at 120 V for 30–60 min (duration adjusted as needed). The resulting DNA bands were visualized and documented using a BIO-RAD gel imaging system.

### Genetic diversity analysis based on designed primers

The amplification products were scored manually. Clear or faint bands were recorded as present (1), while their absence was recorded as (0), generating a binary matrix. This matrix was analyzed using NTSYS-pc version 2.1 to construct a dendrogram based on cluster analysis. Genetic diversity parameters for the cultivated *Dendrobium officinale* accessions from Guizhou, including Nei’s gene diversity index (*H*), Shannon’s information index (*I*), number of polymorphic loci, and percentage of polymorphic loci (PPL), were calculated using POPGENE version 1.32.

### Construction of DNA molecular identity cards

A molecular identity code for each cultivated *Dendrobium officinale* accession was generated by digitally encoding its molecular fingerprint data. The binary matrix (1 for presence, 0 for absence), as the core “fingerprint code”, derived from the SSR amplification products was concatenated in a fixed order of primers and fragment sizes to form a binary string. This core “fingerprint code” was supplemented with additional codes representing the germplasm’s provenance, cultivation region, and cultivation method. The combined “fingerprint code + supplementary code” formed the unique molecular identification number. This alphanumeric string was subsequently converted into a scannable Code128-A barcode using an online barcode generator (http://barcode.cnaidc.com/html/BCGcode128b.php) and into a Quick Response (QR) code using an online QR code generator (https://cli.im/), thereby producing the final DNA molecular identity card.

## Results

### Development of genome-wide SSR molecular markers in *Dendrobium officinale*

**Figure 1:**
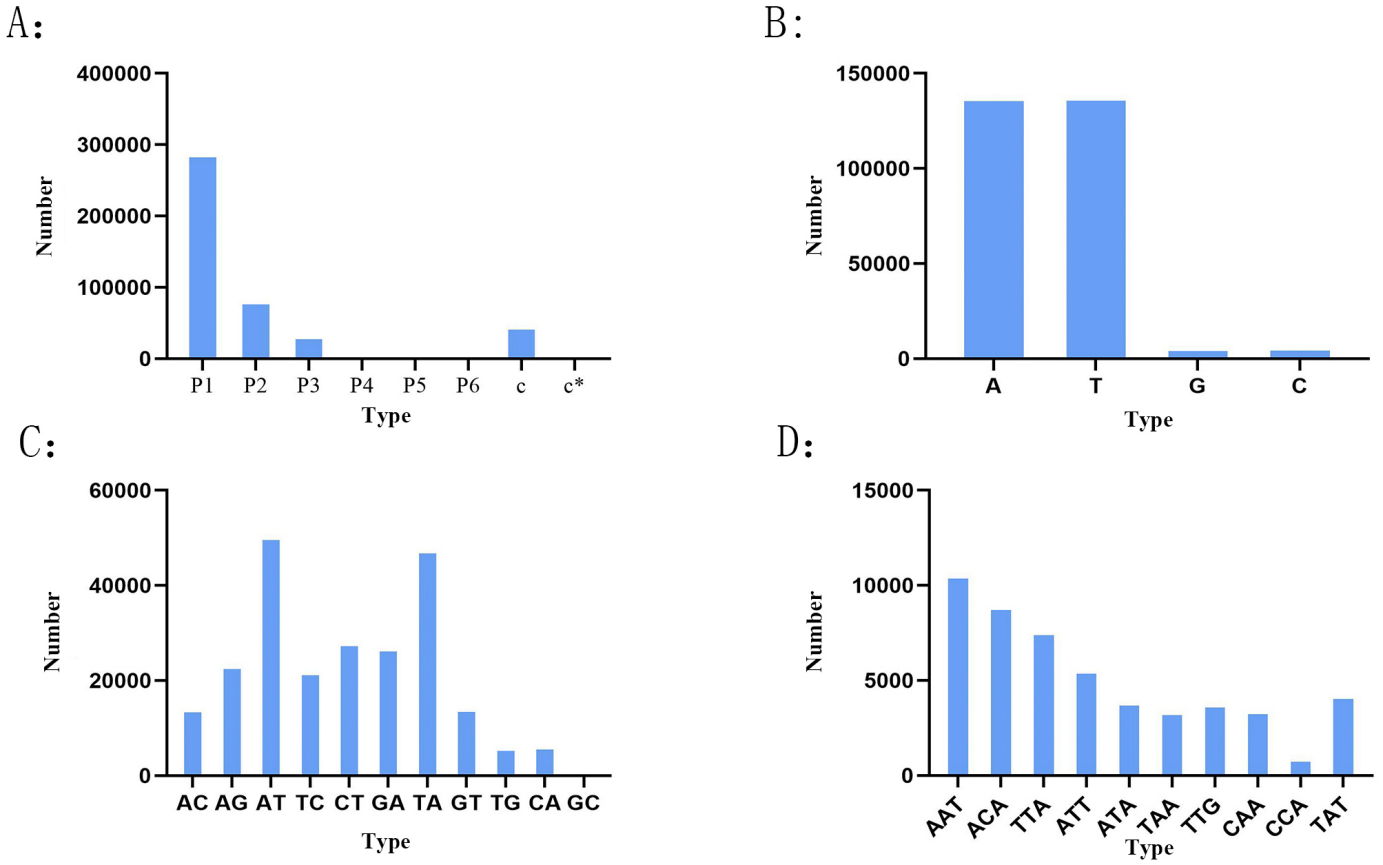
Statistics of different SSR repeat motif types in the whole genome of *D. officinale*. Note: (A) Types and numbers of repeat motifs in the *D. officinale* genome. (B) Major mononucleotide motif types. (C) Major dinucleotide motif types. (D) Major trinucleotide motif types.

A total of 1,361,061 genome-wide SSR loci (Table S1) were identified, from which an average of 136 SSR primer pairs were designed per scaffold, indicating high genome coverage. Statistical analysis revealed seven distinct types of nucleotide SSR repeats, with significant variation in their frequency and abundance (Table 2, Fig. 1A).

**Table 2:** Distribution characteristics of different SSR motif types in the whole genome of *Dendrobium officinale*.

Type	Annotation	Number	Proportion
P1	Mononucleotide motif types	288,312	63.55%
P2	Dinucleotide motif types	81,705	18.01%
P3	Trinucleotide motif types	33,219	7.32%
P4	Tetranucleotide motif types	2,170	0.48%
P5	Pentanucleotide motif types	456	0.10%
P6	Hexanucleotide motif types	198	0.04%
c	Compound motif types^a^	46,601	10.27%
c*	Compound motif types^b^ (interrupted)	1,026	0.23%
Total		453,687	100%

**Notes.**

Compound motif types^a^ SSRs containing multiple motif types in close proximity Compound motif types^b^ (interrupted) Compound SSRs with intervening non-repetitive sequences between motif types

The predominant repeat types were mononucleotide and dinucleotide repeats, collectively accounting for 81.56% of the total SSRs identified. Mononucleotide repeats were the most frequent, constituting 63.51% of all SSRs. Among these, motifs (T) and (A) were overwhelmingly dominant, with 137,806 and 137,747 occurrences respectively, followed by (G) (6,317) and (C) (6,442) (Fig. 1B). Motifs A and T each accounted for approximately 47.77% and 47.81% of the mononucleotide repeats, respectively.

Dinucleotide repeats represented 18.01% of the total. The major dinucleotide motifs were AT/TA, CT, GA, and AG. Specifically, (AT) occurred 17,623 times (21.57% of dinucleotide repeats), (TA) 16,748 times (20.50%), (CT) 9,406 times (11.52%), (GA) 9,006 times (11.03%), and (AG) 7,812 times (9.56%), with (AT) and (TA) being the predominant types (Fig. 1C).

Trinucleotide repeats comprised 8.20% of the total. The most frequent trinucleotide motifs were (AAT) (3,863 occurrences), (ACA) (2,991), and (TTA) (2,822). Other notable motifs included (ATT) (2,064), and (CCA), (CAA), (TTG), (TAA), (ATA), (TAT) with counts ranging from 1,154 to 1,594 (Fig. 1D).

**Figure 2:**
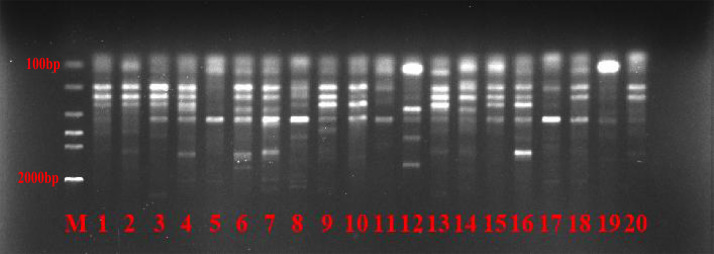
PCR gel electrophoresis pattern of Primer SSR35.

Tetranucleotide, pentanucleotide, and hexanucleotide repeats were relatively scarce, collectively constituting less than 1% of all SSRs. Tetranucleotide repeats accounted for 0.48%, with (TTTA) (319) and (TTTG) (392) as the main types. Pentanucleotide repeats (0.10%) were primarily represented by (AAAAT) (48 occurrences). Hexanucleotide repeats (0.04%) included motifs such as (GTTTAG) (six occurrences) (Fig. 2).

### Genetic diversity analysis of *Dendrobium officinale*

**Figure 3:**
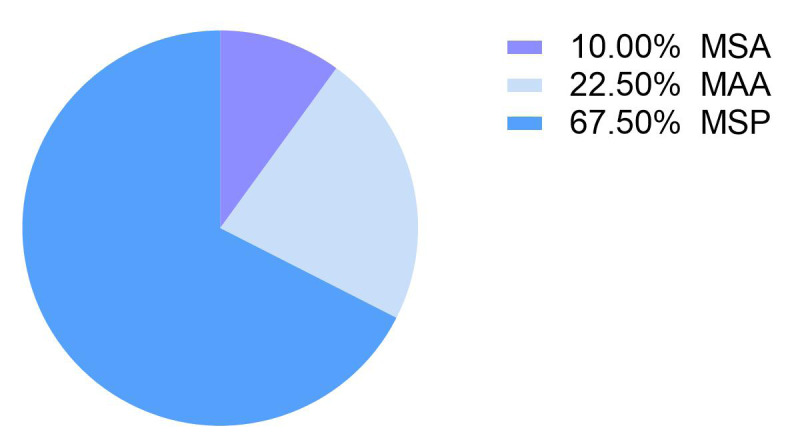
Statistical analysis of PCR results for 27 primer pairs. Note: MSP denotes polymorphic amplification, MAA denotes amplifiable but monomorphic, and MSA denotes no amplification product.

Forty primer pairs were randomly selected (Table 3), of which 27 were polymorphic, 9 produced monomorphic amplification bands, and 4 failed to generate effective amplification products (Fig. 3), resulting in an effective amplification rate of 67.5%. Gel electrophoresis analysis revealed that the 27 SSR primers produced a total of 134 bands, with 127 being polymorphic, yielding a high polymorphism rate of 95%. The number of alleles per primer ranged from 2 to 8, with an average of 4.7 (Tables 4 and 5). Primers SSR15 and SSR20 exhibited the highest polymorphism, each generating 8 effective amplification bands.

**Table 3:** SSR polymorphic primers and their annealing temperatures.

No.	Primer name	Sequence (5′ → 3′)	Genomic location	Annealing temperatures (°C)
1	SSR4	F:5′-TGCCTTTTGAAACACTTCCC-3′	scaffold10:26334-26673	52
		R: 5′-AGCATTTCGCATGATGATTG-3		
2	SSR7	F: 5′-TGAAAGCCACCTGACTTGTG-3′	scaffold1000:87457-87776	50
		R: 5′-ATGTCACATTGCAACCAAGG-3′		
3	SSR8	F: 5′-GCTATGAGAACCACCGAGGA-3′	scaffold1000:118140-118471	52
		R: 5′-ATCATGAACCATGTTGACGC-3′		
4	SSR11	F: 5′-GCCCCCTTAATGCCCTAATA-3′	scaffold10008:23326-23647	52
		R: 5′-CTTCTCGAAACTTATCGCCG-3′		
5	SSR12	F: 5′-ATGGTGAGGCTTCAAACAGG-3′	scaffold10009:21788-22129	52
		R: 5′-CCCCTCTCATTCGTTCTTCT-3′		
6	SSR14	F: 5′-TACCAAGCCACATGAAAGCA-3′	scaffold1001:143018-143347	50
		R: 5′-GCATGATCCTTTTGTTGATACG-3′		
7	SSR15	F: 5′-GTAGGCCGATTCTCCCTCTC-3′	scaffold1001:162834-163187	54
		R: 5′-CGCACCTATAGAGGCTGACC-3′		
8	SSR16	F: 5′-AGAAGCTGGGGACGAATTTT-3′	scaffold10010:26091-26412	54
		R: 5′-CCAAACCCTAGCAACCCTTT-3′		
9	SSR17	F: 5′-TCTTGGGGAGGTAGGATGTG-3′	scaffold10010:35139-35452	54
		R: 5′-TTTTTGAGGTGAAGATGGGC-3		
10	SSR18	F: 5′-TTTTGCCCATGTACATAGCTT-3′	scaffold10012:26594-26929	50
		R: 5′-GTTTGGCCTCCAGGTTTACA-3′		
11	SSR20	F: 5′-AACATAAGCACGTGATGGCA-3′	scaffold4138:5161-5490	52
		R: 5′-AAACCCAAGTGGGGCTTAGT-3′		
12	SSR22	F:5′-CTTTTTGTAGGGTGGGCAAA-3′	scaffold1002:58637-58949	58
		R: 5′-GCGGATCCCCATATAAGTGA-3′		
13	SSR23	F: 5′-ACCAGCTTTTCACCCATTTG-3′	scaffold10029:24414-24727	51
		R: 5′-TTAGCCGGGTTTGAGACTTG-3′		
14	SSR24	F: 5′-CCCAGCAGTGTCACCTATCA-3′	caffold1003:96284-96599	59
		R: 5′-AGGTGGTAAGCTGGGCTAGG-3′		
15	SSR25	F: 5′-CCGGCCAAATGCTTAAAAA-3′	scaffold10033:13080-13470	56
		R: 5′-AATTAAACCACCGCATGCTC-3′		
16	SSR26	F: 5′-TGAAGGCTCGCAACTCATAA-3′	scaffold10042:29322-29637	56
		R: 5′-CAACGCGAAAACAAAACACA-3′		
17	SSR27	F: 5′-CTAGCCAGCTAAGGGAGGCT-3′	scaffold1005:14747-15060	54
		R: 5′-ACCCGAATTGACACCCCTAT-3′		
18	SSR28	F: 5′-GCCCTCTTATTCACCCCTCT-3′	scaffold1006:98333-98674	56
		R: 5′-CGCACTCCGCACCTAATAAT-3′		
19	SSR29	F: 5′-CACCGAACCGAAAACCTAAA-3′	scaffold1007:128676-128998	54
		R: 5′-TTCCCCAGTGTCTCTCTTCC-3′		
20	SSR30	F: 5′-CAAACCATGCTCAATTGCAT-3′	scaffold1008:80396-80741	51
		R: 5′-GCTTACCCTTGCACTTTGGA-3′		
21	SSR31	F: 5′-CTCCAATGGGCCTAATAGCA-3′	scaffold10099:5933-6248	58
		R: 5′-TCTATGTGTTTGCGTTGCGT-3′		
22	SSR32	F: 5′-TATGTTGTTCAATGGCGCTG-3′	scaffold1012:142781-143096	51
		R: 5′-AGGACCCCAAGAGGAAGTCT-3′		
23	SSR33	F: 5′-TGCAAACTGTACAGCACCAA-3′	scaffold1013:121508-121820	50
		R: 5′-TCTGATTTTGGGCAGGGTAG-3′		
24	SSR35	F: 5′-GAGGAACCTCTGCCATGAGA-3′	scaffold10141:29287-29600	51
		R: 5′-TAGGGCATCCTGTTCGTTTT-3′		
25	SSR37	F: 5′-AGCTTTTCAAAACTTGGGTTG-3′	scaffold1015:139261-139611	50
		R: 5′-AAGGGCACTGTAAGGGTGTG-3′		
26	SSR39	F: 5′-GACTCATCGGGGTAGTCCAG-3′	scaffold1017:67635-67980	64
		R: 5′-GTTCCGGGGACTTAAAATGG-3′		
27	SSR40	F: 5′-GTTGAAGGCCGCTATGATGT-3′	scaffold10181:26018-26333	51
		R: 5′-TTTAAGCTTGGCAAAATTTGAT-3′		

**Table 4:** Effective SSR primers and their polymorphism.

No.	Primer	Number of amplified bands	Polymorphic bands	Percentage of polymorphic bands (%)
1	SSR4	3	3	100
2	SSR7	6	5	83
3	SSR8	6	5	83
4	SSR11	7	6	86
5	SSR12	3	3	100
6	SSR14	5	4	80
7	SSR15	8	8	100
8	SSR16	6	5	83
9	SSR17	6	6	100
10	SSR18	5	5	100
11	SSR20	8	8	100
12	SSR22	3	3	100
13	SSR23	4	4	100
14	SSR24	2	1	50
15	SSR25	4	4	100
16	SSR26	2	2	100
17	SSR27	6	6	100
18	SSR28	5	5	100
19	SSR29	5	4	80
20	SSR30	4	4	100
21	SSR31	4	4	100
22	SSR32	7	7	100
23	SSR33	4	4	100
24	SSR35	5	5	100
25	SSR37	3	3	100
26	SSR39	6	6	100
27	SSR40	7	7	100

**Table 5:** Statistics of PCR amplification bands for *Dendrobium officinale* SSR markers.

Item	Value
Number of polymorphic primers	27
Number of polymorphic bands	127
Average number of bands per primer	4.7

Across the 20 *Dendrobium officinale* accessions, a total of 129 polymorphic loci were detected, averaging 4.7 polymorphic loci per primer pair. The percentage of polymorphic loci (PPL) was 96.27%. The values for Shannon’s information index (*I*), Nei’s gene diversity index (*H*), and the average number of alleles (Na) were 0.5205, 0.3491, and 1.9627, respectively. These results indicate that the selected primers possess high polymorphism and that the tested cultivated *Dendrobium officinale* accessions exhibit rich genetic diversity.

**Figure 4:**
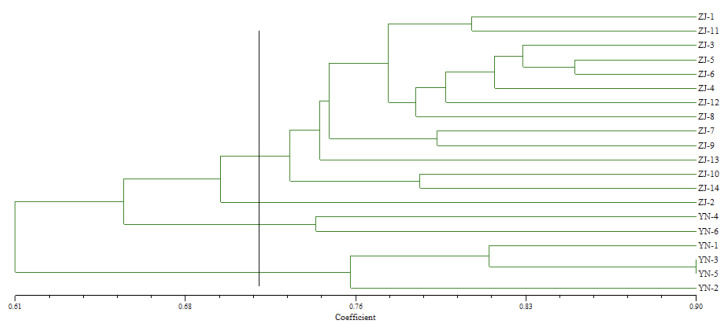
UPGMA dendrogram of cultivated *Dendrobium officinale* from Guizhou Province.

The systematic cluster analysis revealed that, at a similarity coefficient threshold of 0.71, the tested *Dendrobium officinale* accessions could be classified into four major groups (Fig. 4). Group I comprised 13 germplasm resources: ZJ-1, ZJ-2, AL-1, ZJ-3, ZJ-6, ZJ-8, YN-1, ZJ-5, GZ-1, ZJ-4, YN-8, FJ-1, and ZJ-7. This group included eight accessions from Zhejiang, one from Fujian, two from Yunnan, and two from Guizhou. Group II consisted of 13 germplasm resources, with LD-1 representing a locally domesticated *Dendrobium officinale* accession from Luodian County, Guizhou Province. Group III contained two accessions introduced to Dali County (YN-4) and Guangnan County (YN-6) in Yunnan Province. Group IV included four germplasm resources, all of which were the ‘Ruanjiao’ variety of *Dendrobium officinale* from Guangnan County, Yunnan Province.

### Construction of molecular identity cards for cultivated *Dendrobium officinale* in Guizhou

**Figure 5:**
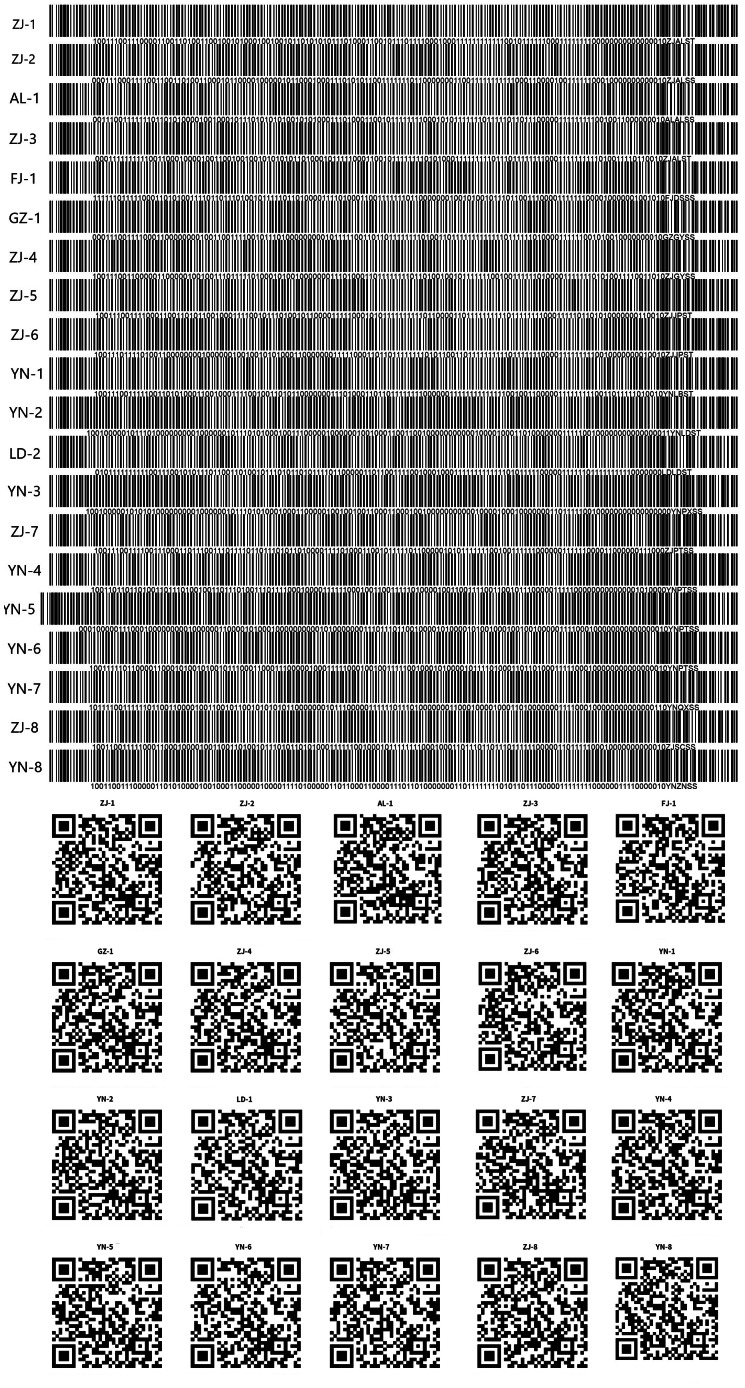
Molecular identity cards for cultivated *Dendrobium officinale* from Guizhou Province.

The molecular fingerprint data of the 20 cultivated *Dendrobium officinale* accessions were digitally encoded. Supplementary codes representing the germplasm’s geographic origin, cultivation region, and cultivation method were appended to create a composite molecular identification number following the “fingerprint code + supplementary code” format (Table 6). This alphanumeric string was subsequently converted into corresponding germplasm-specific QR codes (Fig. 5).

**Table 6:** Molecular identity cards for cultivated *Dendrobium officinale* accessions from Guizhou Province.

Serial no.	Accession ID	Fingerprint code	Supplementary code		
			Origin	Cultivation site	Cultivation method
1	ZJ-1	1001110011100001100110100110010010100010010010110101010111010001100 1011101111000100011111111111100101111110001111111100000000000000010	ZJ	AL	ST
2	ZJ-2	0001110001111001100110100110001011000010000010110001000111010101100 1111111011000000011001111111111100011000010011111100010000000000010	ZJ	AL	SS
3	AL-1	0011100111111101101010000100100010111010101010010101000111010001100 1011111111000101011111110111110110111000001111111110010011000000010	AL	AL	SS
4	ZJ-3	0001111111111001100010000100110010010010101010110100010111110001100 1011111111010100011111111101110111111110001111111111010011110110010	ZJ	AL	ST
5	FJ-1	1111110111110001101010011110110111010010111110110100001111010001100 1111111011000000010010100101110110011100001111111000010000001001010	FJ	DS	SS
6	GZ-1	0001110011110001100000000100110011110010111010000000001011111001101 1011111111010011011111111111110111111010000111111001010010000000010	GZ	GY	SS
7	ZJ-4	1001110011000001100000100100111011111010001010010000000111010001101 1111111011010010010111111100100111111010000111111101010011110011010	ZJ	GY	SS
8	ZJ-5	1001110011110001100110101100100011110010111010010110000111110001010 1111111111011000011011111111110111111110001111101101010000000110010	ZJ	JP	ST
9	ZJ-6	1001110111101001100000000100000010010010101000110000000111110001101 1011111111010110011011111111110111111100001111111110010000000010010	ZJ	JP	ST
10	YN-1	1001110011111001101010001100100011110010011010110000000111010001101 1011111111000000111111111111110010011000001111111110011011111010010	YN	LB	ST
11	YN-2	1001000001011101000000000100000010111010100010011100000100000010010 0011001100100000000000100001000110100000001111100100000000000000011	YN	LD	ST
12	LD-1	0101111111111001110010101110110011010010111010110101111011000001101 1001111001000100011111111111110101111100000111111011111111110000000	LD	LD	ST
13	YN-3	1001000001010101000000000100000010111010100010001100000100100100110 0011000100100000000000100001000100000001101111100100000000000000000	YN	PX	SS
14	ZJ-7	1001110011110011100011011100111011110110111010110100001111010001100 1011111011000001010111111100100111111000000111111000011000000111000	ZJ	PT	SS
15	YN-4	1001101101101001101110100100110111010011101111000100001111110001001 1001111101000010011001111001100101110000011111000000000000001010000	YN	PT	SS
16	YN-5	0001000001110001000000000100000011000010100010000000000101000000011 10111011001000010100001010010001001001000001111000100000000000000010	YN	PT	SS
17	YN-6	1001111101100001100010100101001011100011000111000001000111110001001 0011111001000101000010111101000110110100011111000100000000000000010	YN	PT	SS
18	YN-7	1011110011111101100110000100110010110010101010110000000101110000011 1111011101000000011000100001000110100000001111000100000000000000110	YN	QX	SS
19	ZJ-8	1001100111110001100010000100110011010010111010110101000111111001000 1011111111000100011011101101110111111000001101111100010000000000010	ZJ	SC	SS
20	YN-8	1001100111000001101010000100100011000001000011110100000110110001100 0011101101000000011011111111010110111000001111111100000011110000010	YN	ZN	SS

## Discussion

SSR molecular markers are widely recognized for their high polymorphism, strong specificity, good reproducibility, and codominant inheritance, making them increasingly valuable for genetic diversity assessment, QTL mapping, and genetic map construction for various medicinal plants, such as *Bletilla striata* (Li, 2017), *Paeonia lactiflora* (Ji, 2013), and *Melia azedarach*  (Cai et al., 2021). In this study, genome-wide SSR marker development based on the published genome of *Dendrobium officinale* enabled comprehensive coverage of the genome, providing a robust foundation for subsequent genetic analyses.

The distribution characteristics of SSR motifs in *Dendrobium officinale* revealed a strong AT-rich bias, with mononucleotide repeats dominated exclusively by A/T motifs and dinucleotide repeats enriched for AT/TA. This pattern aligns with observations in other monocot species (Mei et al., 2026) and contrasts with the AAG/CCT preference reported in some dicotyledonous plants (Xiao et al., 2015), reflecting potential lineage-specific genomic features. Notably, the predominance of poly (A/T) mononucleotide repeats, while consistent with the overall AT-rich composition of the genome (Zhong et al., 2025), may pose challenges for marker design, as such repeats can compromise primer specificity and amplification stability. This likely contributed to the 10% non-amplification rate observed among randomly selected primer pairs, underscoring the importance of prioritizing longer repeat motifs (*e.g.*, di- or trinucleotides) in future marker development efforts. Nevertheless, the 67.5% polymorphism detection rate among randomly selected primers confirms that the genome-wide strategy remains efficient and reliable.

The high levels of genetic diversity detected among the cultivated *Dendrobium officinale* accessions from Guizhou, as reflected by the high PPL, H, and I values, are consistent with findings from previous SSR-based studies on this species (Xu et al., 2015). This pattern is likely attributable to the relatively short history of large-scale cultivation (Zheng et al., 2024) and the diverse geographical origins of the germplasm, which include wild populations and locally domesticated materials from multiple regions (Hu et al., 2024). UPGMA clustering further revealed a genetic structure characterized by broad admixture with localized differentiation. The mixed composition of Group I, which comprises accessions from several provinces, suggests historical cross-introduction and mixing of germplasm, whereas the distinct clustering of Group II (Luodian local accession) and Groups III–IV (Yunnan-specific accessions) reflects local domestication or ecological specialization. Such genetic heterogeneity has practical implications: it preserves valuable allelic diversity for future breeding efforts but also poses challenges for variety authentication and quality consistency, highlighting the need for reliable identification tools.

To address these challenges, this study developed a molecular identity card system based on the “fingerprint code + supplementary code” framework. By converting SSR-based digital fingerprints into scannable QR codes, the system enables rapid, field-deployable identification of germplasm. This approach leverages the large information capacity and low cost of QR technology (Li, Su & Yang, 2023), offering a practical solution for variety protection, traceability, and quality control in the *Dendrobium officinale* industry. At the same time, while the 20 accessions analyzed provide a solid proof of concept and reveal substantial genetic diversity, we recognize that this sample size is limited relative to the wide distribution and diverse cultivation sources of *Dendrobium officinale* in Guizhou Province.

In summary, this study established a genome-wide SSR marker set and a corresponding molecular identity card system for cultivated *Dendrobium officinale* in Guizhou. These tools provide a scientific basis for variety identification, traceability, and standardization, supporting the sustainable development of the *Dendrobium officinale* industry.

## Conclusions

This study successfully developed genome-wide SSR markers for *Dendrobium officinale*, revealing high genetic diversity among cultivated accessions in Guizhou. Based on these polymorphic markers, a practical molecular identity card system integrating digital fingerprints with key cultivation data was constructed. This system provides a preliminary framework for germplasm identification, quality traceability, and supports the standardization and sustainable development of the *Dendrobium officinale* industry.

##  Supplemental Information

10.7717/peerj.21558/supp-1 Supplemental Information 1 Misa software configuration file

10.7717/peerj.21558/supp-2 Supplemental Information 2 Perl script that reads raw data, performs initial validation, and formats inputs for the main pipeline

10.7717/peerj.21558/supp-3 Supplemental Information 3 Perl script that aggregates processed results, applies filtering criteria, and writes the final output tables

## Additional Information and Declarations

### Data Availability

The following information was supplied regarding data availability: The code is available in the Supplemental Files. The Misa software configuration file, Perl script, and Genome-wide SSR molecular marker developed in this research (Table S1) have been uploaded to the science database: - https://doi.org/10.57760/sciencedb.34283
The raw sequence data are available at NCBI: ASM160598v2, GCA_001605985.2.
